# Effectiveness of Wearable Digital Therapeutics in Improving Sleep Outcomes Among Individuals With Insomnia: Systematic Review and Meta-Analysis of Randomized Controlled Trials

**DOI:** 10.2196/93496

**Published:** 2026-09-11

**Authors:** Wenhui Zhu, Mingming Chen, Yixuan Guo, Bin Zhang, Chunliu Luo

**Affiliations:** 1 School of Nursing, Jinan University, Guangzhou, Guangdong, China; 2 First Affiliated Hospital of Jinan University, 613 Huangpu Avenue West, Guangzhou, Guangdong, 510630, China, 86 18926202718

**Keywords:** insomnia symptoms, sleep problems, wearable electronic devices, digital health, meta-analysis

## Abstract

**Background:**

Wearable devices are increasingly used for sleep monitoring and as adjunctive treatment. Existing meta-analyses mostly pool composite digital therapies and rarely isolate stand-alone wearables or distinguish between objective and subjective end points. Whether stand-alone wearable interventions improve sleep outcomes in adults with insomnia, and which factors moderate treatment heterogeneity, remains unclear.

**Objective:**

This study aims to evaluate the effectiveness of wearable digital interventions on sleep outcomes in adults with insomnia versus control strategies and explore moderators of effectiveness, including device-wearing position, intervention duration, and control type, using meta-regression.

**Methods:**

This systematic review and meta-analysis was conducted in accordance with the PRISMA (Preferred Reporting Items for Systematic Reviews and Meta‑Analyses) 2020 statement and the PRISMA-S (Preferred Reporting Items for Systematic Reviews and Meta‑Analyses Literature Search Extension) guideline. Five electronic databases and clinical trial registries were searched from inception to May 18, 2026. Eligible studies were randomized controlled trials (RCTs) evaluating wearable digital interventions in adults with insomnia compared with sham, waitlist, usual care, or active control conditions and had an intervention duration of at least 1 week. Study screening, data extraction, and risk-of-bias assessment were carried out independently by 2 reviewers. Pooled estimates were calculated using a restricted maximum likelihood random-effects model with the Hartung-Knapp-Sidik-Jonkman correction. Heterogeneity was assessed using the *I*² statistic, and 95% prediction intervals (PIs) were calculated for the primary analyses. The certainty of evidence was rated using the GRADE (Grading of Recommendations, Assessment, Development, and Evaluation) approach.

**Results:**

Sixteen RCTs (N=910) were included. Wearable digital interventions were associated with a significant reduction in objective sleep-onset latency (SOL; mean difference [MD] −4.52, 95% CI −8.38 to −0.67, PI −9.52 to 0.47 min) and a significant improvement in subjective sleep efficiency (SE; MD 2.00%, 95% CI 1.90%‐2.11%, PI 1.85%‐2.15%). Subjective total sleep time (TST) also showed a significant increase (MD 19.11, 95% CI 2.98‐35.24, PI −16.20 to 54.43 minutes). Meta-regression showed that control type, intervention duration, and device location did not explain the heterogeneity of the insomnia severity index (ISI) (*R*^²^=0). Sensitivity analysis confirmed the robustness of pooled ISI estimates, and an Egger test indicated no small-study effects (*P*=.07). Certainty of evidence ranged from moderate to high.

**Conclusions:**

Wearable digital interventions provide selective benefits for objective SOL, subjective SE, and subjective TST in adults with insomnia, with no improvement in overall ISI. Despite statistically significant effects on several sleep parameters, wide PIs, substantial heterogeneity, and limited study numbers indicate preliminary, nonconclusive findings. Wearables should be viewed as affordable adjunctive tools requiring further validation, not substitutes for first-line cognitive behavioral therapy for insomnia. Large-scale, long-term RCTs with standardized protocols and patient-level external validation are required to consolidate the evidence base.

## Introduction

Insomnia, defined as persistent difficulty in initiating or maintaining sleep accompanied by clinically significant daytime impairment [1,2], remains one of the most prevalent sleep disorders worldwide. Epidemiological estimates indicate that approximately 10% to 20% of the general adult population meet diagnostic criteria for insomnia disorder [1,3], with prevalence rising markedly with age, and women being 1.3 to 1.7 times more likely to be affected than men across all age groups [4,5]. Beyond its immediate impact on sleep quality, insomnia constitutes a major risk factor for various mental disorders, such as depression, anxiety, and alcohol dependence, and is additionally associated with diminished quality of life and increased all-cause mortality [4,6-9], thereby imposing a substantial economic and health care burden on society [10]. In the United States alone, the direct costs of insomnia-related medical care, combined with indirect costs from workplace productivity loss, absenteeism, and accidents, have been estimated to exceed US $100 billion annually [11,12]. In aging societies across East Asia and Europe, this burden is further exacerbated by the disproportionately high prevalence of insomnia among older adults [13,14] and the corresponding rise in demand for chronic disease management [15]. Given this profound public health impact, identifying safe, effective, and scalable interventions for insomnia remains a critical priority for health care systems globally.

The management of insomnia encompasses psychological, behavioral, pharmacological, and complementary approaches [3,9]. Among these, cognitive behavioral therapy for insomnia (CBT-I) is recommended as first-line treatment on the basis of robust empirical evidence [2,5]. However, its widespread implementation is severely constrained. The global shortage of trained CBT-I therapists [16], together with high per-session costs (which average US $260 in the United States [17]) and marked geographic disparities in service availability [17], means that the vast majority of patients with insomnia rarely receive guideline-recommended behavioral treatment [2,3,5]. Pharmacological treatments, including benzodiazepine receptor agonists and Z-drugs, provide short-term symptomatic relief but entail substantial risks with long-term use [18], including physical dependence, tolerance, rebound insomnia on discontinuation, cognitive impairment, next-day sedation, and increased fall risk, especially in older adults [19]. Consequently, clinical guidelines universally recommend limiting hypnotic use to the lowest effective dose and the shortest necessary duration, with pharmacotherapy generally restricted to short‑term use of no more than 4 to 5 weeks [20]. The persistent gap between high treatment demand and limited access to evidence-based nonpharmacological therapies highlights the need for scalable alternatives that do not require the infrastructure and workforce associated with traditional CBT-I.

In recent years, digital therapeutics, particularly wearable devices, have emerged as a rapidly advancing frontier in sleep medicine [21-23]. Adoption of wearable sleep technology, including smartwatches, wristbands, head-mounted electroencephalography (EEG) systems, and therapeutic filtered-light glasses, has grown substantially, driven by increasing public awareness of sleep health and advances in miniaturized sensing technology [24,25]. These devices enable continuous, real-time physiological monitoring (eg, heart rate variability, actigraphy [26]) and can provide active therapeutic functions such as acoustic stimulation, transcranial electrical stimulation, and biofeedback-based sleep retraining [2,26-28]. Nevertheless, the available synthesized evidence on the efficacy of stand-alone wearable devices for insomnia remains limited.

Several earlier systematic reviews have sought to synthesize evidence in this field. For instance, Jung et al [8] examined mobile apps for insomnia but did not specifically focus on wearable devices used as stand-alone interventions. Bai et al [29] and Hwang et al [30] assessed digital CBT-I programs rather than the wearable hardware itself. Baron et al [31] and Glazer Baron et al [32] centered their reviews on measurement rather than on therapeutic efficacy. Lai et al [33] reviewed wearable-delivered interventions but pooled heterogeneous populations and did not include any studies published after December 2021. Critically, however, few prior reviews have systematically distinguished between objectively measured sleep parameters and patient-reported subjective sleep outcomes.

Critically, this review was designed to address several knowledge gaps that were not fully addressed in previous meta-analyses. First, rather than aggregating composite digital therapies (eg, wearables combined with app-guided psychotherapy), we focused exclusively on stand-alone wearable interventions to isolate their pure therapeutic effect. Second, we explicitly stratified outcomes into objective sleep parameters (measured by actigraphy or polysomnography) and subjective patient-reported outcomes, given that these 2 domains frequently yield discrepant findings in insomnia research and carry distinct clinical implications. Third, we performed targeted meta-regression to quantitatively investigate sources of heterogeneity in insomnia severity scores, an approach not systematically applied in prior reviews. Fourth, we used the confidence distribution approach proposed by Nagashima et al [34] to compute prediction intervals (PIs), thereby going beyond average pooled estimates to provide a more reliable estimate of the true effect distribution across different clinical settings and populations.

Taken together, the existing literature remains inadequate, owing to fragmentation across technologies, heterogeneity in the conceptualization and measurement of sleep outcomes, and a scarcity of evaluations that isolate the stand-alone effect of wearable hardware while simultaneously distinguishing objective end points from subjective ones and exploring sources of heterogeneity. This review addresses these limitations by concentrating exclusively on stand-alone wearable devices, categorizing outcomes according to objective and subjective measures, and using more conservative statistical methods, including the Hartung-Knapp-Sidik-Jonkman (HKSJ) approach [35] and Nagashima-corrected PIs [34]. Accordingly, the aim of this systematic review and meta-analysis was to evaluate the therapeutic efficacy of stand-alone wearable digital interventions on objective and subjective sleep outcomes relative to various control conditions in adults with insomnia.

## Methods

### Protocol and Guidance

This systematic review and meta-analysis was conducted in accordance with the PRISMA (Preferred Reporting Items for Systematic Reviews and Meta-Analyses) 2020 statement [36] and was registered prospectively with PROSPERO (International Prospective Register of Systematic Reviews). The PRISMA 2020 expanded checklist is provided in Checklist 1. No deviations from the registered protocol occurred during the study.

### Eligibility Criteria

We included randomized controlled trials (RCTs) [37] that used wearable-delivered interventions for adults with insomnia. Eligible participants were adults with an insomnia severity index (ISI) score >7 or those meeting the diagnostic criteria for insomnia disorder as defined by established international classification systems (eg, the *Diagnostic and Statistical Manual of Mental Disorders* or the International Classification of Sleep Disorders). Wearable digital devices (eg, smartwatches, wristbands, and rings) were defined as devices equipped with sleep monitoring as well as active therapeutic functions (eg, acoustic stimulation, filtered light glasses). The intervention was required to be compared against a control condition such as usual care, a sham device, or a waitlist control.

Exclusion criteria were as follows: (1) acute or critical illnesses in participants, (2) intervention duration of less than 1 week, (3) unavailability of full text of the article, and (4) duplicate publications or studies with overlapping data.

### Information Sources

We searched the following electronic databases from inception to April 30, 2025, without language restrictions: PubMed, Embase, Web of Science, PsycINFO, and the Cochrane Central Register of Controlled Trials. The search was updated on May 18, 2026, to capture newly published studies. The full search strategies for each database are provided in Multimedia Appendix 1.

### Search Strategy

The search was reported according to the PRISMA-S (Preferred Reporting Items for Systematic Reviews and Meta-Analyses literature search extension) guideline [38]. We searched PubMed, Embase, Web of Science, PsycINFO, and the Cochrane Library from inception to April 30, 2025 (updated May 18, 2026). The search combined controlled vocabulary (MeSH in PubMed/Cochrane, Emtree in Embase) and free-text terms for 3 domains: wearable devices (eg, “wearable electronic devices,” “actigraphy,” “smartwatch”), insomnia (eg, “insomnia,” “sleep initiation and maintenance disorders”), and RCTs (eg, “randomized controlled trial,” “RCT”). No language or date restrictions were applied. The detailed search strategies for each database are provided in Multimedia Appendix 1. The PRISMA-S checklist is provided in Checklist 2. Backward and forward citation searches of included studies were also performed.

No multidatabase searching, independent registry searches, online browsing, contacting authors, use of published search filters, adaptations from prior reviews, or formal peer review were performed. Each database was searched separately.

### Selection Process

Two independent reviewers (WZ and MC) screened the titles, abstracts, and full texts of all retrieved articles. Studies assessed as potentially relevant or unclear at the title and abstract stage were obtained in full text and reevaluated. Any disagreements between the reviewers were resolved through discussion or by consultation with a third investigator (YG).

### Data Collection Process

Data were extracted independently by 2 reviewers (WZ and MC) using a standardized data extraction form. Any disagreements were resolved through discussion or consultation with a third reviewer (YG). When data were missing or unclear, we contacted the corresponding authors of the original studies via email. No automation tools were used in the data collection process.

### Data Items

For each included study, we extracted the primary outcome (ISI), secondary outcomes (objective sleep parameters: total sleep time [TST], sleep-onset latency [SOL], wake after sleep onset [WASO], sleep efficiency [SE]; and subjective sleep parameters: Pittsburgh Sleep Quality Index [PSQI], subjective TST, SOL, WASO, SE, Epworth Sleepiness Scale [ESS], and single-item sleep quality), and other variables (first author, year, country, design, participant characteristics, intervention details, comparator, attrition, intention-to-treat or missing data methods, trial registration, and funding).

### Study Risk-of-Bias Assessment

The Cochrane Risk-of-Bias 2.0 (RoB 2.0) tool for randomized trials was used to evaluate the methodological quality of the included studies across 5 bias domains. Assessments were carried out independently by 2 reviewers (WZ and MC).

### Effect Measures

For continuous outcomes (ISI, TST, SOL, WASO, SE, ESS, PSQI, and sleep quality), the effect measure was the mean difference (MD) with a 95% CI. When different scales were used for the same construct (eg, different sleep quality measures), we planned to use the standardized mean difference (SMD; Hedges *g*). However, all included studies reported outcomes on the same or directly convertible metrics, so MD was used throughout. All included trials used identical measurement units for each sleep parameter (eg, minutes for SOL, TST, and WASO and percentages for SE), enabling direct pooling with MD rather than SMD. Between-study differences in absolute values reflect clinical and demographic heterogeneity in study populations rather than any inconsistency in outcome scaling.

### Synthesis Methods

Studies were grouped by outcome domain (ISI, objective sleep parameters, and subjective sleep parameters). Postintervention means and SDs were extracted; when only change scores were reported, the postintervention SD was calculated using a correlation coefficient of 0.5 [39]. Meta-analyses were performed using a random-effects model with the HKSJ correction (metafor package in R [R Foundation for Statistical Computing]) to reduce the risk of inflated type I errors [36,40], with heterogeneity assessed using *I*² statistics and *Q* tests. Nagashima-corrected 95% PIs [34] were calculated for all pooled outcomes and displayed in forest plots. To explore heterogeneity, meta-regression was conducted only for ISI with 3 prespecified moderators (control type, intervention duration, and device-wearing position). Sensitivity analyses included leave-one-out analysis for ISI and rerunning all meta-analyses after the updated literature search.

### Reporting Bias Assessment

Funnel plots and Egger regression test were used to examine small-study effects (not publication bias directly). For outcomes with fewer than 10 studies, these tests were not performed due to limited statistical power. No trim-and-fill or other adjustment methods were applied. No other reporting bias assessments were conducted.

### Certainty Assessment

The certainty of evidence for each outcome was evaluated using the GRADE (Grading of Recommendations, Assessment, Development, and Evaluation) approach. Two reviewers (WZ and MC) independently rated the evidence across 5 domains: risk of bias, inconsistency, indirectness, imprecision, and publication bias. Disagreements were resolved by consensus. The overall certainty was graded as high, moderate, low, or very low. Outcomes with fewer than 3 studies were not graded.

## Results

### Study Selection

**Figure 1.**
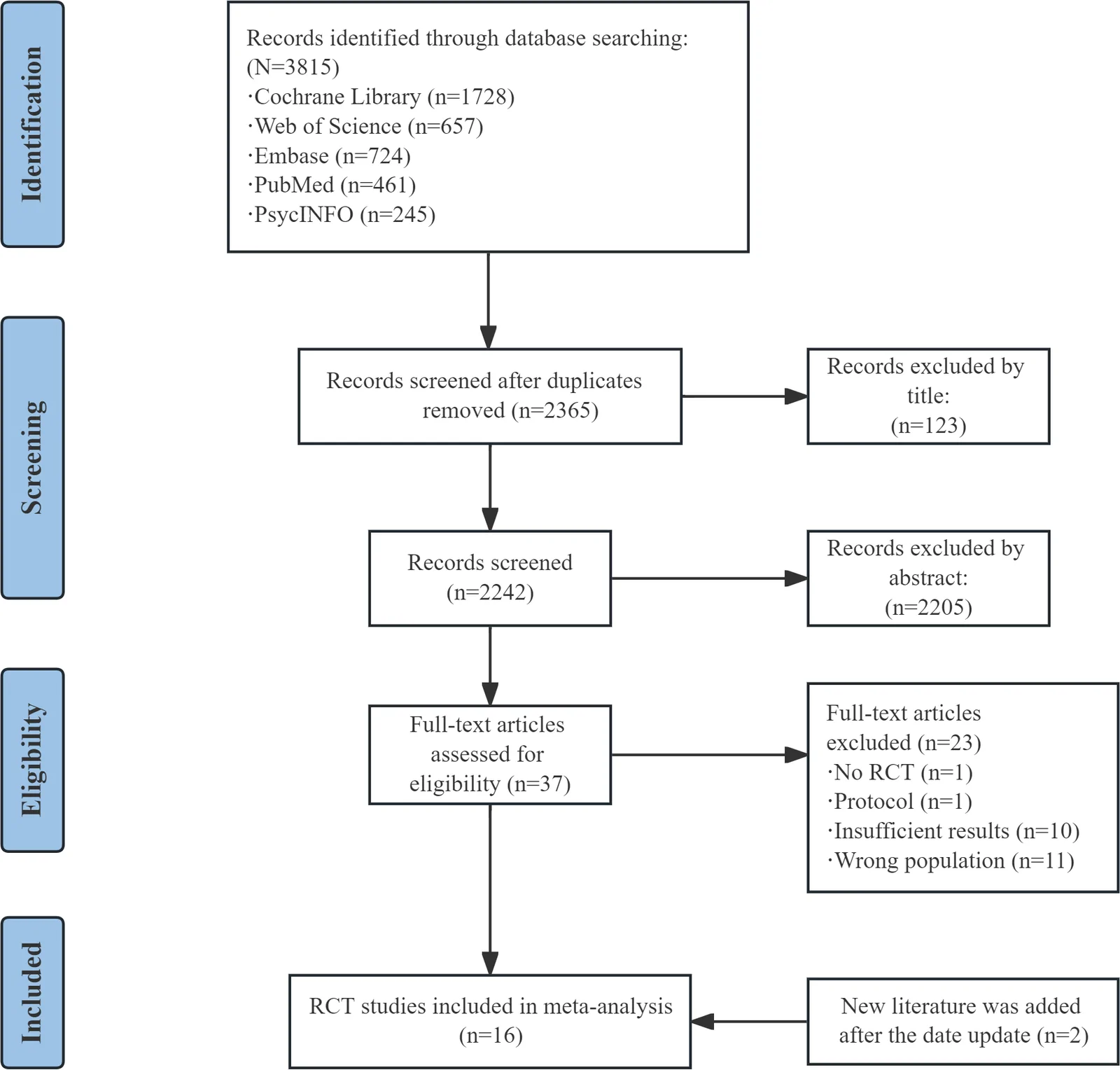
Flowchart for study selection. RCT: randomized controlled trial.

Of the 3815 records identified through database searching, 3799 were excluded after the removal of duplicates and the exclusion of irrelevant studies. All studies included independent participants, and no double-counting occurred. We updated the literature search on May 18, 2026, adding 2 new RCTs, and conducted a reanalysis of the statistics. The remaining 16 RCTs [41-56] met the eligibility criteria and were included in the final analysis. The study selection process is summarized in the PRISMA flow diagram (Figure 1).

### Study Characteristics

Most studies reported low-to-moderate attrition rates. However, Aji et al [42] reported a high dropout rate (40/91, 44.0% among app initiators), with significantly more dropouts in the control group than in the intervention group, which the authors attributed to the fully digital, unsupervised design, and lack of face-to-face contact. Zabrecky et al [56] reported a dropout rate of 23.1% (9/39), mainly due to scheduling conflicts (n=4), inability to tolerate MRI scanning (n=2), and unspecified reasons (n=3), with no separate reporting by randomization group. For the remaining 8 studies, attrition rates ranged from 0% to 16%, and reasons for dropout were mostly unrelated to the intervention.

Among the 16 included RCTs, a total of 910 participants were enrolled across all studies, with sample sizes ranging from 14 to 152 per trial. A summary of the characteristics of the included studies is presented in Table 1. The publication years of the included studies spanned from 2017 to 2026; 11 [41-43,47-52,54,56] reported the ISI, 9 [45,47-52,54-56] reported TST, and 12 [41,43,45-52,54,55] contained objective sleep data, while 10 [41-43,46-49,51,52,55] included subjective sleep outcomes. The comparators consisted of sham interventions [41,43-49,52,53,55], waitlist [50,56], usual care [42,51], and active control [54]. Interventions were delivered via wearable devices: head-mounted devices [41,44-46,48,50,52,54], therapeutic filtered light glasses [47,49], neck-worn devices [43,53], ear-worn devices [55], and wristbands [42,51,56]. The intervention duration ranged from 2 weeks to 3 months. For all outcomes, posttreatment values were extracted to calculate effect sizes, as detailed in the “Methods” section.

**Table 1.** Characteristics of the included studies.

Author, year, country	Design	Population	Sample size	Intervention	Control	Outcomes measures	Attrition (%)	ITT^a^/MDM^b^	Protocol/registration	Grant
Aji et al [42], 2022, Australia	2-arm RCT^c^	ISI^d^≥15 and PSQI^e^ global>5 (42.6±10.7)	T^f^: 128 I^g^: 62 C^h^: 66	Wearable technology (Fitbit Charge 2)	dBTi^i^ only	ISI, daytime sleepiness (ESS^j^)	44	No/No	No/Yes	Yes
Anderson et al [43], 2025, United States	2-arm RCT	ISI≥15 (40.4±12.1)	T: 58 I: 27 C: 31	Evolv28 (wearable neckband device)	Sham device	Primary: ISI Secondary: sleep duration, WASO^k^ (min), SE^l^ (%) Objective: (actigraphy) TST^m^ (min), SE (%), WASO (min)	8.47	Yes/Yes	Yes/Yes	Yes
Bressler et al [44], 2023, United States	RCT	ISI≥21 and PSQI global>5 (33.0±6.6)	T: 48 I: 24 C: 24	Elemind Neuromodulation Device (a wearable EEG^n^)	Sham device	Sleep physiology metrics: sleep stage scoring, SOL^o^, phase tracking accuracy of alpha oscillations (8‐12 Hz) Subjective: (sleep diary) bedtime, wake time, sleep quality	0	No/No	No/No	Yes
Chen et al [45], 2026, China	2-arm RCT	PSQI>7 (I: 20.85±2.2; C: 20.44±1.7)	T: 20 I: 10 C: 10	cTBS^p^	Sham device	Objective (actigraphy): SOL, TST, TA^q^, SFI^r^	0	Yes/No	No/No	Yes
Curry et al [46], 2024, United Kingdom	2-arm RCT	ISI≥15 (40.8±13.5)	T: 126 I: 61 C: 65	Modius Sleep device	Sham device	Primary: change in ISI score Secondary: PSQI, RAND 36-Item Short Form Survey, caffeine diaries	15.65	Yes/Yes	Yes/Yes	Yes
Esaki et al [47], 2020, Japan	2-arm RCT	Bipolar disorder ISI≥8 (I: 44.1±11.8; C: 41.1±10.4)	T: 43 I: 21 C: 22	Blue-blocking orange glasses	Clear placebo glasses	Objective (actigraphy): SE, SOL, WASO, TST Subjective: VAS^s^, ISI, MEQ^t^	14.0	Yes/No	Yes/Yes	Yes
He et al [48], 2024, China	4-arm RCT	ISI>7 and PSQI global >5 (67.68±4.98)	T: 152 I1: 38 I2: 38 I3: 38 C: 38	I1: TC^u^+active rTMS^v^ I2: TC+sham rTMS I3: TC alone	Low-intensity PE	Primary: ISI Secondary: Objective (actigraphy): TST (min), SOL (min), SE (%), WASO (min), number of awakenings, average wake time per awakening. Subjective (sleep diary): daytime sleepiness: Chinese version of the ESS	9.21	Yes/Yes	Yes/Yes	Yes
Janků et al [49], 2020, Czech Republic	2-arm RCT	Diagnosed with insomnia (48.1±16.1)	T: 30 I: 15 C: 15	UVEX S1933X orange glasses	UVEX S1900 clear glasses	Subjective (sleep diaries): SOL (min), TST (min), WASO (min), SE (%), SQ^w^ Objective (actigraphy): SOL (min), TST (min), WASO (min), SE (%)	14.29	No/No	No/No	Yes
Jeon and Choi [50], 2017, South Korea	2-arm RCT	ISI≥15 and PSQI global ≥5 (I: 23.5±1.73; C: 25.6±2.88)	T: 14 I: 5 C: 9	Neurofeedback therapy (Procomp 5)	Waiting only	Objective: TST (min), SL^x^ (min), SE (%) (Smart Wearable Device) Subjective: insomnia symptom severity (ISI), PSQI, ESS, Presleep Arousal Scale (sleep diary)	0	Yes/Yes	No/No	Yes
Kang et al [51], 2017, South Korea	2-arm RCT	Insomnia disorder (45.1±9.8)	T: 19 I: 10 C: 9	Wearable sleep tracker (Fitbit Charge HR)	CBT-I^y^ and app only	Objective: SE (%) (actigraphy) Subjective: sleep quality (PSQI), insomnia symptom severity (ISI), SE (%) (PSQI), TST (min), SL (min), TIB^z^ (min), SE (%) (sleep diary)	0	No/No	No/No	Yes
Kennedy et al [53], 2023, United States	2-arm RCT	ISI≥8 (51.18±10.50)	T: 30 I: 15 C: 15	CeraZ Technologies LLC	Sham device	Objective (oura ring): SE (%), TST (min), TIB (min), SOL (min) Subjective (sleep diaries): SOL (min), sleep quality, Karolinska Sleepiness Scale scores	0	No/No	No/Yes	Yes
Lee et al [41], 2024, Korea	2-arm RCT	ISI≥8 BDI-II=20 (I: 49.40±11.76; C: 48.67±12.90)	T: 38 I: 20 C: 18	MAVE device	Sham device	Subjective: PSS, BDI-II^aa^, ISI, PSQI, STAI^ab^, WHOQOL-BREF^ac^ Objective: qEEG^ad^, ACTH, cortisol, BDNF^ae^	16.33	No/No	Yes/Yes	Yes
Liu et al [52], 2025, China	2-arm RCT	Sleep disorders, experiencing MCI^af^ without dementia (67.9±4.6)	T: 110 I: 55 C: 55	rTMS+TC	Sham rTMS+ TC	Objective: (actigraphy) SE, SOL, WASO, TST Subjective: PSQI, ESS, HAMA^ag^, HAMD^ah^, MoCA^ai^	6.4	Yes/No	Yes/Yes	Yes
Simons et al [54], 2024, United States	2-arm RCT	ISI≥8 (MI^aj^: 52; MC^ak^: 50.5)	T: 24 I: 12 C:12	SDR-tES^al^ (wearable device)	Active control	Primary: SOL Secondary: TST, WASO, ISI, SE, STAI (state subform)	0	No/Yes	No/Yes	Yes
Yeom et al [55], 2025, South Korea	2-arm RCT	ICSD-3^am^, PSQI≥5 (I: 31.9±12.3; C: 29.4±8.0)	T: 40 I: 20 C: 20	Transcutaneous auricular vagus nerve stimulation	Sham device	Objective: (Fitbit) TST Subjective: PSQI, ISI, WHOQOL-BREF	2.5	Yes/Yes	Yes/Yes	Yes
Zabrecky et al [56], 2020, United States	2-arm RCT	Insomnia disorder (I: 43.3±19.6; C: 40.8±1.6)	T: 30 I: 19 C: 11	VibrAcoustic Wellness System	Waiting only	Objective: (actigraphy) neurological assessment: resting state functional magnetic resonance imaging Subjective: ISI	23.08	No/No	No/No	Yes

a ITT: intention-to-treat analysis.

b MDM: missing data management.

c RCT: randomized controlled trial.

d ISI: insomnia severity index.

e PSQI: Pittsburgh Sleep Quality Index.

f T: total.

g I: intervention.

h C: control.

i dBTi: digital behavioral therapy for insomnia.

j ESS: Epworth Sleepiness Scale.

k WASO: wake after sleep onset.

l SE: sleep efficiency.

m TST: total sleep time.

n EEG: electroencephalography.

o SOL: sleep-onset latency.

p cTBS: continuous theta burst stimulation.

q TA: time awake.

r SFI: sleep fragmentation index.

s VAS: visual analog scale.

t MEQ: Morningness–Eveningness Questionnaire.

u TC: Tai Chi.

v rTMS: repetitive transcranial magnetic stimulation.

w SQ: sleep quality.

x SL: sleep latency.

y CBT-I: cognitive behavioral therapy for insomnia.

z TIB: time in bed.

aa BDI-II: Beck Depression Inventory-II.

ab STAI: State Trait Anxiety Index.

ac WHOQOL-BREF: World Health Organization Quality of Life‑BREF.

ad qEEG: Quantitative electroencephalography.

ae BDNF: brain‑derived neurotrophic factor.

af MCI: mild cognitive impairment.

ag HAMA: Hamilton Anxiety Rating Scale.

ah HAMD: Hamilton Depression Rating Scale.

ai MoCA: Montreal Cognitive Assessment.

aj MI: median intervention.

ak MC: median control.

al SDR-tES: short duration repetitive transcranial electric stimulation.

am ICSD-3: International Classification of Sleep Disorders‑Third Edition.

### Results of Syntheses

### ISI

**Figure 2.**
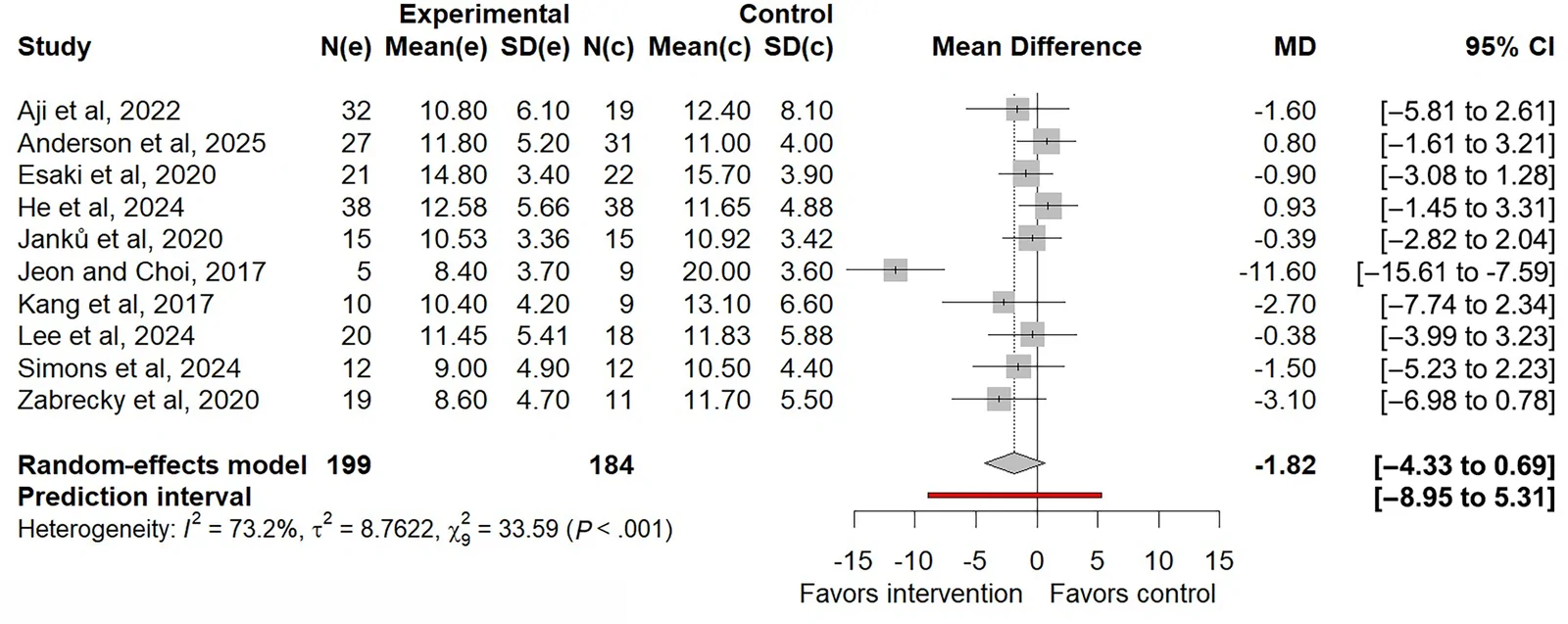
Forest plot of insomnia severity index for wearable treatment vs control across 10 randomized controlled trials (adults with insomnia, published between 2017 and 2025). Pooled estimates were calculated using the Hartung‑Knapp‑Sidik‑Jonkman random-effects model, with the Nagashima correction for prediction intervals [41-43,47-51,54,56]. MD: mean difference.

ISI scores were reported in 10 studies. The pooled random-effects estimate, using the HKSJ correction, showed no significant reduction in ISI scores (MD −1.82, 95% CI −4.33 to 0.69, PI −8.95 to 5.31), with substantial between-study heterogeneity (*I*²=73.2%; Figure 2).

### Objective Sleep Outcomes

**Figure 3.**
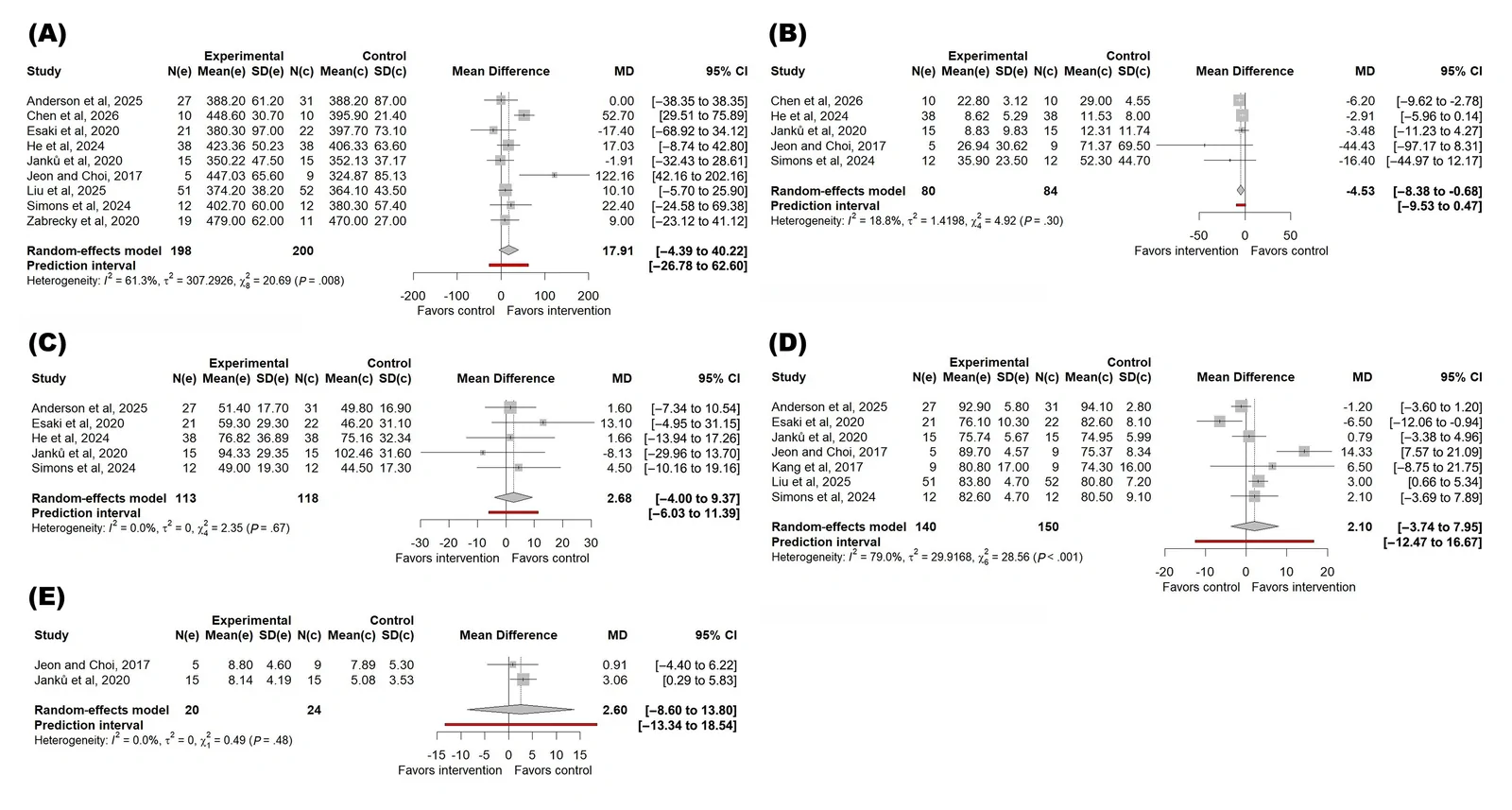
Forest plots of objective sleep outcomes for the wearable intervention vs control. Pooled estimates were calculated using the Hartung‑Knapp‑Sidik‑Jonkman random-effects model, with the Nagashima correction applied to the prediction intervals. (A) Total sleep time (*k*=9): nonsignificant (mean difference [MD] 17.91, 95% CI −4.39 to 40.22 min; *I*²=61.3%). (B) Sleep-onset latency (*k*=5): significant reduction (MD −4.52, 95% CI −8.38 to −0.67 min; I²=18.8%). (C) Wake after sleep onset (*k*=5): nonsignificant (MD 2.68, 95% CI −4.00 to 9.37 min; *I*²=0%). (D) Sleep efficiency (*k*=7): nonsignificant (MD 2.10%, 95% CI −3.74% to 7.95%; *I*²=79.0%). (E) Epworth Sleepiness Scale (*k*=2): nonsignificant (MD 2.60, 95% CI −8.60 to 13.80 points; *I*²=0%) [43,45,47-52,54,56].

A series of meta-analyses was conducted for objective TST (n=9), SOL (n=5), WASO (n=5), SE (n=7), and the ESS (n=2), as shown in Figure 3. For TST, the random-effects meta-analysis using the HKSJ correction showed a nonsignificant pooled MD of 17.91 (95% CI −4.39 to 40.22; PI −26.78 to 62.60) minutes. For SOL, a significant reduction was observed, with a pooled MD of −4.52 (95% CI −8.38 to −0.67; PI −9.52 to 0.47) minutes, indicating that wearable interventions shortened the time to fall asleep compared with controls. The meta-analyses found that wearable interventions did not have a statistically significant effect on WASO (MD 2.68, 95% CI −4.00 to 9.37, PI −6.30 to 11.39 minutes), SE (MD 2.10%, 95% CI −3.74% to 7.95%; PI −12.47% to 16.67%), and ESS (MD 2.60, 95% CI −8.60 to 13.80; PI −13.34 to 18.54 points).

### Subjective Sleep Outcomes

**Figure 4.**
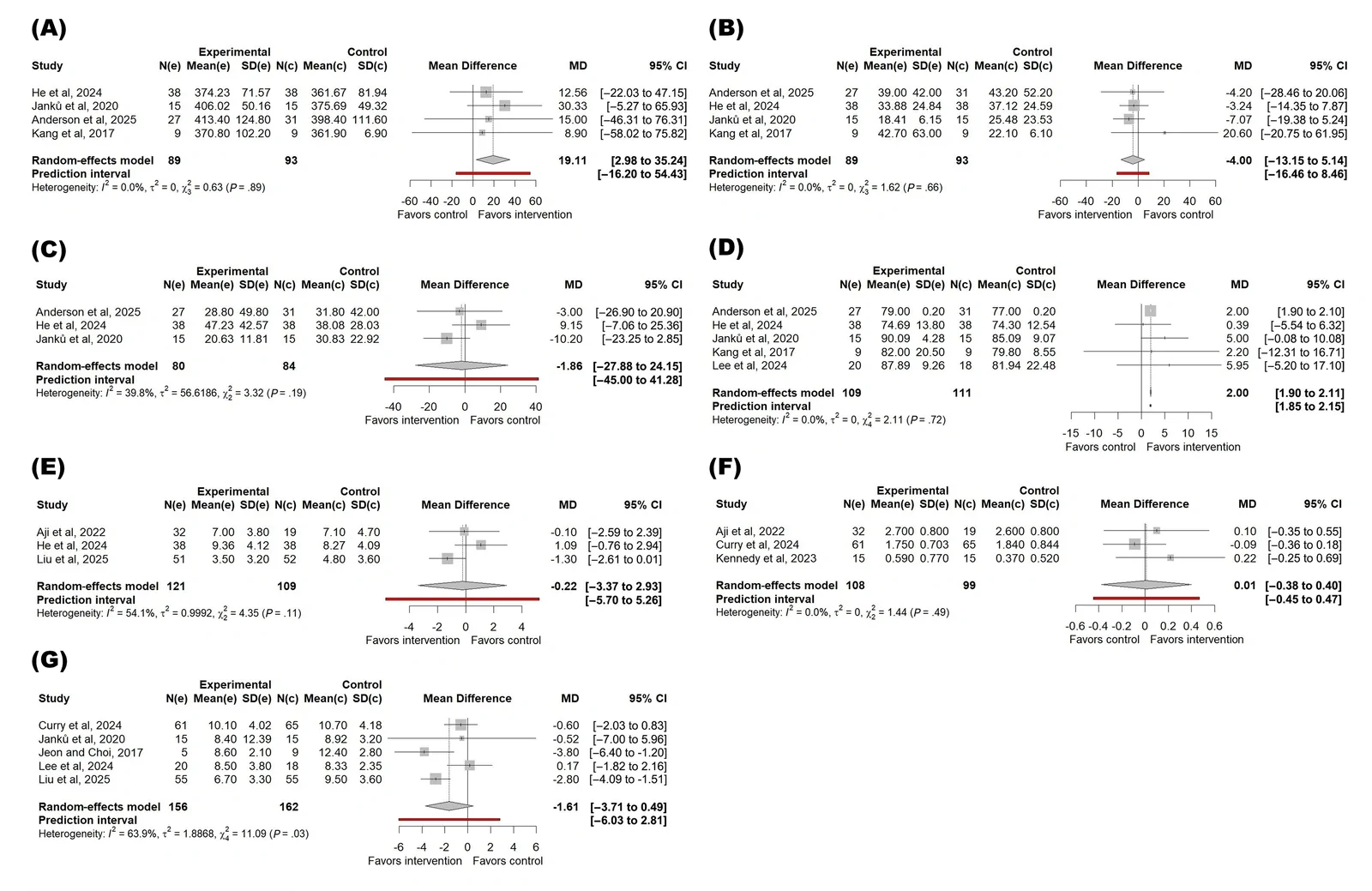
Forest plots of subjective sleep outcomes for wearable interventions vs control in adults with insomnia (randomized controlled trials, published between 2017 and 2025). Pooled estimates were calculated using the Hartung‑Knapp‑Sidik‑Jonkman random-effects model, with the Nagashima correction applied to prediction intervals (PIs). (A) Total sleep time (*k*=4): significant increase (MD 19.11, 95% CI 2.98 to 35.24 min), but the PI crossed zero (PI −16.20 to 54.43), indicating variable effects across trials. (B) Sleep-onset latency (*k*=4): nonsignificant (MD −4.0, 95% CI −13.15 to 5.14 min). (C) Wake after sleep onset (*k*=3): nonsignificant (MD −1.86, 95% CI −27.88 to 24.15 min). (D) Sleep efficiency (*k*=4): significant improvement (MD 2.00%, 95% CI 1.90% to 2.11%). (E) Epworth Sleepiness Scale (*k*=3): nonsignificant (MD −0.22, 95% CI −3.37 to 2.93 points). (F) Sleep quality (*k*=3): nonsignificant (MD 0.01 points, 95% CI −0.38 to 0.40 points). (G) Pittsburgh Sleep Quality Index (*k*=5): nonsignificant reduction (MD −1.61, 95% CI −3.71 to 0.49 points) [41-43,46,48,50-53].

Meta-analyses were conducted for subjective sleep outcomes, including TST (n=4), SOL (n=4), WASO (n=3), SE (n=4), ESS (n=3), sleep quality (n=3), and PSQI (n=5), as shown in Figure 4. Subjective SE showed a small beneficial effect (MD 2.00%, 95% CI 1.90%‐2.11%; PI 1.85%‐2.15%) in the wearable group compared with controls. Subjective TST was significantly improved (MD 19.11, 95% CI 2.98‐35.24; PI −16.20 to 54.43 min), suggesting inconsistent efficacy across individual trials despite the statistically significant pooled overall effect. For the remaining subjective sleep end points, all 95% CIs crossed the null value and demonstrated no statistically meaningful intervention benefits: subjective SOL (MD −4.0, 95% CI −13.15 to 5.14; PI −16.46 to 8.46 min), subjective WASO (MD −1.86, 95% CI −27.88 to 24.15; PI −8.95 to 5.31 min), subjective ESS (MD −0.22, 95% CI −3.37 to 2.93, PI −5.70 to 5.26 points), subjective sleep quality (MD 0.01, 95% CI −0.38 to 0.40, PI −0.45 to 0.47 points), and PSQI (MD −1.61, 95% CI −3.71 to 0.49; PI −6.03 to 2.81 points).

### Investigation of Heterogeneity: Meta-Regression

Subgroup analyses were predefined for multiple sleep outcomes; however, most end points had fewer than 5 trials, precluding reliable subgroup pooling. Accordingly, mixed-effects meta-regression with Hartung-Knapp adjustment was selected as an alternative method to investigate between-study heterogeneity. To identify potential sources of substantial between-study heterogeneity for the ISI, we performed mixed-effects meta-regression with 3 predefined study-level moderators: control type, intervention duration, and device-wearing position. A multivariate model incorporating all 3 covariates was constructed first, followed by 3 separate univariate meta-regression analyses for individual moderator assessment.

In the multivariate regression model, the omnibus *F* test for combined moderators was nonsignificant (*F*_3,6_=0.26; *P*=.85), and these covariates collectively explained 0.00% of interstudy heterogeneity (*R*^²^=0.00%). Marked unexplained residual heterogeneity remained after adjustment (residual *I*²=83.48%; residual heterogeneity *Q*‑statistic*, Q*_E_=29.08; *df*=6 for *Q*_E_; *P*<.001). None of the 3 moderators yielded statistically significant regression coefficients (control type: *P*=.83; intervention duration: *P*=.58; device‑wearing position: *P*=.60; Table 2). Consistent findings were observed in subsequent univariate analyses: control type (*F*_1,8_=0.29; *P*=.61), intervention duration (*F*_1,8_=0.60; *P*=.46), and device-wearing position (*F*_1,8_=0.10; *P*=.76) failed to alter pooled effect estimates. All univariate models returned *R*^²^ of 0.00%, with residual *I*^²^ ranging from 78.60% to 80.96%. Overall, the 3 prespecified clinical factors could not account for the high heterogeneity of pooled ISI results.

**Table 2.** Meta-regression analysis of potential moderators for the insomnia severity index (ISI).

Moderator	Coefficient	Standard error	*t* test (*df*)	*P* value	95% CI	*R*² (%)^a^	Residual *I*² (%)^b^
Multivariate model							
Control type	0.749	3.28	0.228 (6)	.83	−7.276 to 8.773	0	83.48
Intervention duration	1.883	3.235	0.582 (6)	.58	−6.033 to 9.800	0	83.48
Device-wearing position	1.723	3.124	0.552 (6)	.60	−5.922 to 9.368	0	83.48
Univariate model							
Control type	1.281	2.396	0.535 (8)	.61	−4.244 to 6.806	0	79.81
Intervention duration	1.807	2.329	0.776 (8)	.46	−3.564 to 7.177	0	78.6
Device-wearing position	0.85	2.674	0.318 (8)	.76	−5.316 to 7.016	0	80.96

a *R*²: percentage of between-study heterogeneity explained by moderators.

b *I*²: residual unexplained heterogeneity.

### RoB in Studies

**Figure 5.**
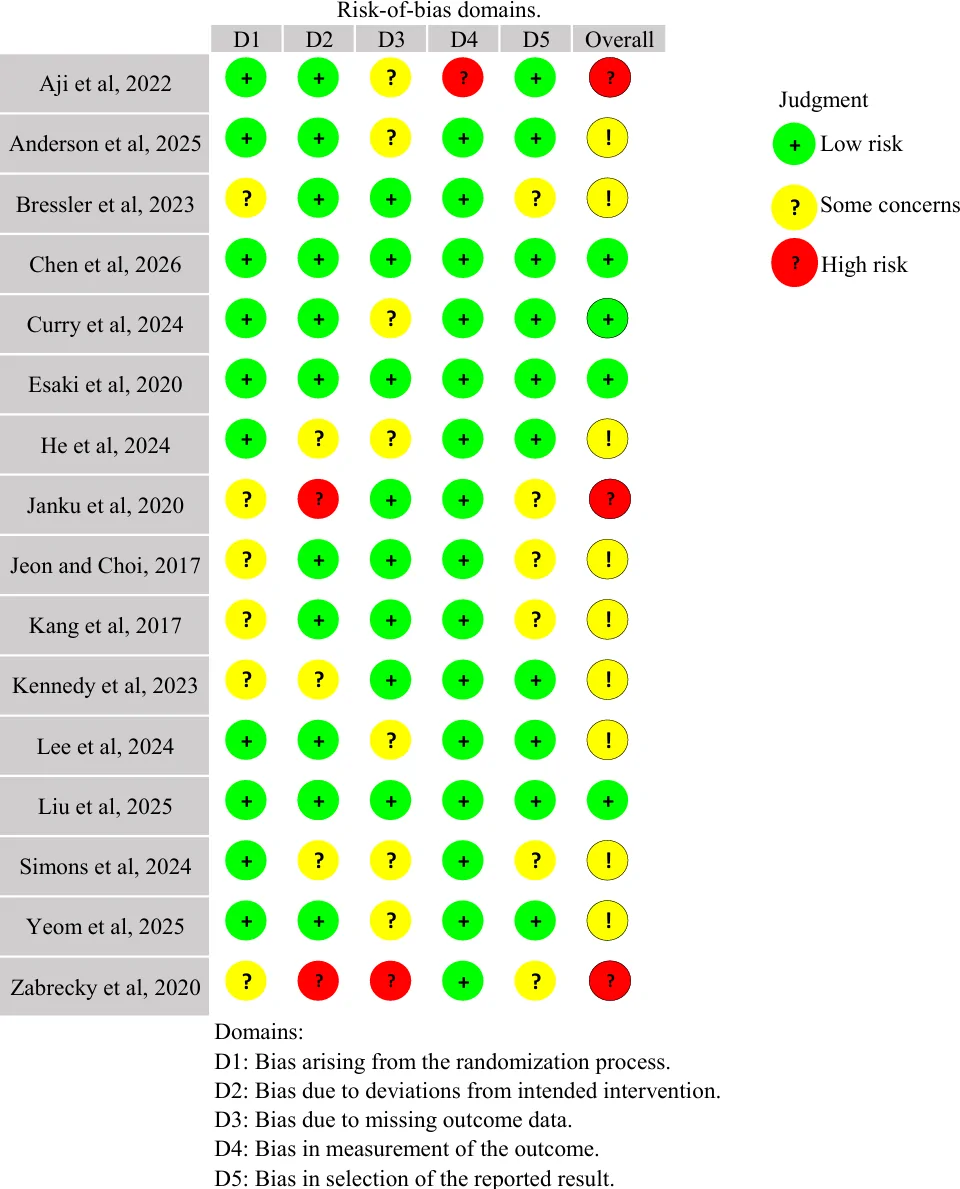
Risk-of-bias (RoB) summary: assessment of the included randomized controlled trials using the Cochrane RoB 2.0 tool [41-56].

The RoB in the included RCTs was independently assessed by 2 reviewers using the Cochrane RoB 2.0 tool. Any disagreements were resolved by consensus or consultation with a third investigator. Overall, 4 studies were classified as having a low RoB, 9 as having an unclear RoB, and 3 as having a high RoB (Figure 5).

### Reporting Biases

For the ISI outcome, which included 10 RCTs, we generated funnel plots and applied the random-effects regression test (the regtest function in the metafor package) to assess small-study effects rather than publication bias. We found borderline nonsignificant funnel asymmetry (*z*=−1.85; *P*=.06). No quantitative tests for small-study effects were undertaken for the remaining outcomes, as fewer than 10 studies were available, leading to low statistical power.

### Sensitivity Analysis

Leave-one-out sensitivity analysis was performed under the HKSJ random-effects model to verify the robustness of the pooled ISI estimate. Sequential exclusion of each individual study did not substantially alter the direction and magnitude of the pooled effect. Excluding the small-sample trial conducted by Jeon and Choi [50] eliminated between-study heterogeneity, yet the overall effect remained consistent, which further confirmed the stability of our primary findings.

### Certainty of Evidence

The GRADE rating results (Table 3) showed that 2 outcomes were rated as moderate certainty due to imprecision, and 3 outcomes were rated as high certainty. Outcomes with fewer than 3 studies were not graded.

**Table 3.** GRADE^a^ summary-of-findings table for wearable digital therapy in adults with insomnia.

Certainty assessment							Patients, n/N (%)		Effect	Certainty
Number of studies	Study design	Risk of bias	Inconsistency	Indirectness	Imprecision	Other considerations	Wearable device	Control	Absolute (95% CI)	
Insomnia severity index										
10	Randomized trials	Not serious	Serious^b^	Not serious	Serious^c^	None	199/383 (52.0)	184/383 (48.0)	MD^d^ –1.82 (–4.33 to 0.69)	⨁⨁◯◯ Low^b^^,c^
Pittsburgh Sleep Quality Index										
5	Randomized trials	Not serious	Serious^e^	Not serious	Serious^f^	None	156/318 (49.1)	162/318 (50.9)	MD –1.61 (–3.71 to 0.49)	⨁⨁◯◯ Low^e^^,f^
Objective total sleep time										
9	Randomized trials	Not serious	Serious^g^	Not serious	Serious^h^	None	198/398 (49.7)	200/398 (50.3)	MD 17.91 (−4.39 to 40.22)	⨁⨁◯◯ Low^g^^,h^
Subjective total sleep time										
4	Randomized trials	Not serious	Not serious	Not serious	Not serious	None	89/182 (48.9)	93/182 (51.1)	MD 19.11 (2.98 to 35.24)	⨁⨁⨁⨁ High
Objective sleep efficiency										
7	Randomized trials	Not serious	Serious^i^	Not serious	Serious^j^	None	140/290 (48.3)	150/290 (51.7)	MD 2.10 (−3.74 to 7.95)	⨁⨁◯◯ Low^i^^,j^
Subjective sleep efficiency										
4	Randomized trials	Not serious	Not serious	Not serious	Not serious	None	109/220 (49.5)	111/220 (50.5)	MD 2.00 (1.90 to 2.11)	⨁⨁⨁⨁ High
Objective sleep-onset latency										
5	Randomized trials	Not serious	Not serious	Not serious	Not serious	None	80/164 (48.8)	84/264 (31.8)	MD −4.52 (−8.38 to −0.67)	⨁⨁⨁⨁ High
Subjective sleep-onset latency										
4	Randomized trials	Not serious	Not serious	Not serious	Serious^k^	None	89/182 (48.9)	93/182 (51.1)	MD –4.0 (−13.15 to 5.14)	⨁⨁⨁◯ Moderate^k^
Objective WASO^l^										
5	Randomized trials	Not serious	Not serious	Not serious	Serious^m^	None	113/231 (48.9)	118/231 (51.1)	MD 2.68 (−4.00 to 9.37)	⨁⨁⨁◯ Moderate^m^

a GRADE: Grading of Recommendations, Assessment, Development, and Evaluation.

b Downgraded one level for substantial heterogeneity (*I*²=73.2%, *P*<.001).

c Downgraded one level for wide CI crossing the null (95% CI −4.33 to 0.69) and broad prediction interval.

d MD: mean difference.

e Downgraded one level for moderate-to-substantial heterogeneity (*I*²=58.3%; *P*=.026).

f Downgraded one level for CI crossing the null (95% CI −2.73 to 0.45) and broad prediction interval.

g Downgraded one level for moderate heterogeneity (*I*²=61.3%; *P*=.008).

h Downgraded one level for CI crossing the null (95% CI −4.39 to 40.22) and very broad prediction interval (−26.78 to 62.60).

i Downgraded one level for substantial heterogeneity (*I*²=79.0%; *P*<.0001), largely driven by a single outlier (Jeon and Choi [50]) with an opposite effect direction; sensitivity analysis excluding this study reduced heterogeneity to 39.9% but the pooled estimate remained nonsignificant.

j Downgraded one level for wide CI crossing the null (95% CI −3.74 to 7.95) and very broad prediction interval (−12.47 to 16.67), indicating substantial uncertainty in the true-effect estimate.

k Downgraded one level for serious imprecision. The wide 95% CI (−13.15 to 5.14) crosses the null, including both beneficial and harmful effects, so a clinically meaningful intervention effect cannot be confirmed.

l WASO: wake after sleep onset.

m Downgraded one level for CI crossing the null (−4.00 to 9.37) and being relatively wide, indicating that the true effect could range from a modest reduction to a clinically meaningful increase in WASO.

## Discussion

This systematic review and meta-analysis of 16 RCTs encompassing 910 participants indicates that stand-alone wearable digital interventions are associated with small yet statistically significant improvements in objective SOL, subjective SE, and subjective TST among adults with insomnia. Nevertheless, these interventions demonstrate no significant benefit for global insomnia severity as measured by the ISI. Through the application of the HKSJ framework with Nagashima-corrected PIs, this study provides more robust effect estimates than prior reviews. Although pooled estimates favor wearable interventions for these specific parameters, the wide 95% PIs suggest that the effect in any given future clinical setting could range from meaningful benefit to negligible or no effect. This implies that while wearable interventions are validated at the population level for certain sleep outcomes, their real-world effectiveness is highly dependent on context [57-59]. This finding underscores the importance of exploring sources of heterogeneity, a prespecified objective of this review. The certainty of the evidence was rated as moderate to low using GRADE [39], reflecting consistent effect direction for specific outcomes while also acknowledging recognized methodological limitations, particularly the inability to blind participants and outcome assessors in wearable device trials.

The selective efficacy pattern observed in this review suggests that wearable devices target specific physiological and perceptual sleep domains rather than the multifaceted cognitive and behavioral pathology that underlies insomnia disorder [60,61]. This interpretation aligns with the theoretical understanding that insomnia is not merely a disorder of sleep physiology but also involves maladaptive cognitions, conditioned arousal [62], and behavioral factors [63] that require targeted psychotherapeutic intervention [2]. Hardware-based biofeedback and neuromodulation delivered via wearable devices may facilitate physiological de-arousal and shorten sleep onset [54,64,65]; however, they do not directly address the perpetuating mechanisms of insomnia, such as sleep-related worry, conditioned bed arousal, and irregular sleep schedules, that CBT-I is designed to modify [2,60,66]. The certainty of these findings, however, must be tempered by the underlying evidence quality: objective SOL and subjective SE were rated as high certainty by GRADE, providing confidence that wearable devices produce measurable improvements in these specific parameters. By contrast, subjective TST, though statistically significant, was based on only 4 trials and exhibited a wide PI. Its GRADE rating was high primarily due to consistency across the limited number of studies, a finding that should be interpreted with caution given the small evidence base. Furthermore, the overall RoB assessment (Figure 5) showed that most included trials had unclear or high RoB across multiple domains. Insufficient blinding of participants and personnel, an inherent challenge in wearable device trials, was the most common concern [67] and may have inflated effect estimates for subjective outcomes that rely on self-report. Empirical evidence from meta-epidemiological studies shows that nonblinded assessors exaggerate effect estimates for subjective outcomes by 29% (95% CI, 8‑45) on average [67], and a systematic review reported a 68% exaggeration [68]. These findings suggest that the true effects of wearable interventions on subjective sleep outcomes may be smaller than observed. Additionally, several studies had high or unclear RoB due to incomplete outcome data, such as attrition rates exceeding 20% in some trials, a lack of intention-to-treat analysis, or inadequate reporting of randomization and allocation concealment. These methodological limitations, especially when combined with small sample sizes and variable adherence, suggest that the observed effects may be overestimated. Consequently, our more modest and selective findings, compared with larger effect sizes reported for comprehensive digital CBT-I, likely reflect the genuine stand-alone contribution of wearable hardware rather than the confounded psychotherapeutic effects present in prior pooled analyses.

The subjective-objective discrepancy identified in our analysis is consistent with the well-documented phenomenon of sleep misperception in insomnia. Patients with insomnia often underestimate their sleep duration and overestimate their wakefulness relative to objective recordings, a pattern known as paradoxical insomnia or sleep-state misperception [69,70]. Our finding that objective SOL shortened, whereas subjective TST exhibited considerable between-study variability implies that wearable devices may induce genuine physiological changes in sleep onset without necessarily correcting the perceptual distortions that characterize insomnia [61]. This observation has direct implications for patient counseling: individuals using wearable devices may experience faster objective sleep onset but may not perceive a corresponding improvement in overall sleep quality or duration, which could lead to continued dissatisfaction. Ahn et al [71] further showed that greater sleep-wake state discrepancy was associated with poorer treatment response, indicating that unresolved perceptual distortions may undermine perceived treatment benefit even when objective sleep metrics improve.

In comparison with existing syntheses, the modest therapeutic impact of stand-alone wearables becomes apparent. Unlike digital CBT-I programs evaluated in previous reviews [29,30], which address both sleep physiology and the cognitive behavioral perpetuating factors of insomnia, our exclusive focus on wearable hardware reveals improvements limited to specific sleep parameters rather than global insomnia resolution. Reviews of consumer sleep technology [31,32] have largely focused on measurement rather than therapeutic efficacy and did not quantitatively compare different device types or intervention durations. Lai et al [33] previously conducted a review that specifically examined wearable-delivered interventions but pooled heterogeneous populations and did not target insomnia disorder exclusively. Our findings advance this prior work by demonstrating that, in a homogeneous insomnia population, wearables produce selective benefits that are significant for sleep onset and perceived efficiency but not for global insomnia severity, thereby clarifying their appropriate role as adjunctive rather than primary interventions.

From a methodological perspective, the application of Nagashima-corrected PIs [34] offers a more nuanced interpretation than conventional confidence intervals alone. For example, although objective SOL demonstrated a statistically significant average effect, the PI suggested that the true effect in future individual settings could range from meaningful benefit to negligible change or even no effect. Similarly, the significant average improvement in subjective TST was accompanied by a PI that crossed zero, implying that the perceived benefit is inconsistent across settings and may depend on unmeasured moderators, such as baseline sleep misperception severity, device engagement, or placebo expectations. This distinction between average effects and effect distributions is of paramount importance for clinical decision-making: a statistically significant confidence interval confirms that an intervention works on average across trials, whereas the PI reveals whether it is likely to work in a particular clinical setting or for a given individual patient [58].

The high unexplained heterogeneity for ISI (residual *I*²=83.48% after meta-regression) is noteworthy. The inability of control type, intervention duration, and device-wearing position to explain this heterogeneity implies that the true variability in treatment effects may be attributable to factors that were not captured in published aggregate data. One such factor may be baseline objective sleep duration. Bathgate et al [72] showed that patients with insomnia with objectively measured short sleep duration (<6 h) exhibited a significantly blunted response to CBT-I compared with those with normal sleep duration (≥6 h), suggesting that patients with a more biologically severe insomnia phenotype may be less responsive to behavioral interventions in general. If baseline objective sleep duration similarly moderates response to wearable devices, its absence from the included trials may account for at least part of the unexplained heterogeneity we observed. Other potential sources include baseline insomnia severity, comorbid mental health conditions, concomitant sleep medication use, and individual differences in sleep physiology and perception. Head-worn devices typically use EEG, which provides a direct and physiologically precise measure of sleep and may enable more targeted neuromodulation [73,74]. In contrast, wrist-worn actigraphy, while convenient, is prone to overestimating sleep by misclassifying quiet wakefulness as sleep [75]. Furthermore, inconsistent operational standards for wearable device calibration across different manufacturers could also contribute to measurement variation [27,76]. Of note, although device-wearing position differed across included trials, our prespecified meta-regression confirmed that such hardware divergence was not a statistically significant source of heterogeneity for ISI.

The RoB and GRADE assessments provide important context for our findings. Of the 16 included trials, 9 had an unclear overall RoB and 3 had a high risk, largely attributable to the inherent difficulty of blinding participants and personnel in wearable device trials [39]; consequently, the pooled estimates may be subject to performance and detection bias. The direction of such bias likely favors the intervention group, meaning that the true effects may be smaller than observed. According to the GRADE assessment, 3 outcomes were rated as high certainty, 2 as moderate certainty, and 4 as low certainty (Table 3). The high-certainty outcomes provide confidence that wearable devices produce measurable improvements in these specific parameters. By contrast, the moderate- and low-certainty ratings for the remaining outcomes, resulting from imprecision, inconsistency, or both, indicate that the true effects may differ materially from the pooled estimates, and clinical recommendations derived from these outcomes should be viewed as conditional rather than definitive.

Several limitations should be considered when interpreting the findings of this review. First, the modest number of studies contributing to several pooled outcomes (fewer than 5 trials for roughly half of the analyses) constitutes an important limitation that restricts the statistical reliability of the corresponding estimates and precludes meaningful meta-regression for these end points. The inability to obtain individual patient data further prevented the exploration of patient-level predictors of treatment response, which likely represent key sources of the unexplained heterogeneity we observed for ISI [57]. Future studies with larger numbers of trials and standardized reporting of individual-level characteristics are needed to confirm the robustness of these findings. Second, the predominance of unclear or high RoB across most included trials, primarily due to insufficient blinding as an inherent challenge in wearable device trials, may have inflated effect estimates for subjective outcomes that rely on self-report. Moreover, most trials had intervention durations of 3 months or less, so the long-term efficacy and sustainability of wearable interventions beyond this timeframe remain unknown [77]. Future trials should incorporate longer follow-up periods and more rigorous blinding procedures where feasible. Third, most participants were of East Asian or North American origin, with limited representation from other regions; females predominated, and mean ages varied substantially. Therefore, the findings may not be generalizable to male-only populations, younger adults, or non-Asian and non-Western populations. Future research should prioritize diverse and representative samples to enhance generalizability.

These findings have several practical clinical implications. Consistent with the objective-subjective outcome discrepancy we identified, wearable devices should be considered an adjunct rather than a substitute for first-line CBT-I. This view is supported by Spina et al [61], who found that providing feedback on wearable-measured sleep data reduced insomnia severity (*d*=0.51), although this effect was modest compared with established CBT-I effect sizes (*d*=0.85). These devices provide objective sleep biofeedback and facilitate continuous monitoring. Head-mounted EEG equipment enables precise physiological measurement, whereas wrist-worn trackers may overestimate sleep duration [27,76]; therefore, head-worn devices may be better suited for improving objective sleep parameters, although cost and tolerability must be carefully considered in clinical practice. The selective efficacy pattern suggests that wearables may be most appropriate for patients with predominant sleep-onset difficulties or those who perceive their sleep as inefficient. Clinicians should consider baseline patient characteristics, including insomnia subtype, comorbidity, and treatment expectations, when recommending wearables, and they should manage expectations concerning the likely magnitude and consistency of benefit. For future research, large and long-term RCTs are needed to establish long-term efficacy. Novel trials should collect detailed participant baseline data to address unexplained heterogeneity and should include daytime function and quality-of-life outcomes. The integration of wearable real-time feedback with digital CBT-I represents a promising avenue for individualized insomnia management.

This meta-analysis has several strengths. It strictly followed Cochrane systematic review guidance, and the literature search was updated as of May 2026 with 2 newly added RCTs. Stratified analyses that separated objective and subjective sleep indicators helped clarify the distinct discrepancy in efficacy between physiological recordings and patient-reported outcomes. Meta-regression was used instead of fragmented subgroup analyses to systematically explore prespecified confounding moderators contributing to ISI heterogeneity, consistent with current methodological standards. Comprehensive methodological assessments, including RoB evaluation, small-study effect detection via Egger test, leave-one-out sensitivity analysis, and GRADE certainty ratings, further strengthened the reliability and transparency of the results.

In conclusion, the evidence from this review positions stand-alone wearable devices as a potentially valuable but limited tool in the insomnia therapeutic landscape. Their greatest promise likely lies not in replacing first-line therapies but in serving as an objective feedback mechanism and monitoring adjunct that could enhance patient engagement and enable treatment personalization. To move beyond the current paradigm of population-level efficacy but context-dependent effectiveness, the field must pivot from simple efficacy trials toward implementation science. Future research should prioritize the development and evaluation of hybrid models that combine wearable biofeedback with digital CBT-I, invest in large-scale, long-term pragmatic trials across diverse populations, and leverage individual patient data to clarify the heterogeneity of response. By doing so, it becomes possible to unlock the full potential of wearable therapeutics, shifting from a one-size-fits-all approach toward a stratified insomnia management pathway that aligns technological capabilities with patients’ specific pathophysiological and psychological profiles.

## Supplementary material

10.2196/93496 Multimedia Appendix 1 Full search strategies for each database.

10.2196/93496 Checklist 1 PRISMA checklist.

10.2196/93496 Checklist 2 PRISMA-S checklist.

## Abbreviations

EEG: electroencephalography
ESS: Epworth Sleepiness Scale
GRADE: Grading of Recommendations, Assessment, Development, and Evaluation
ISI: insomnia severity index
MD: mean difference
PI: prediction interval
PRISMA: Preferred Reporting Items for Systematic Reviews and Meta‑Analyses
PRISMA-S: Preferred Reporting Items for Systematic Reviews and Meta‑Analyses Literature Search Extension
PROSPERO: International Prospective Register of Systematic Reviews
PSQI: Pittsburgh Sleep Quality Index
RCT: randomized controlled trial
RoB: risk of bias
SE: sleep efficiency
SMD: standardized mean difference
SOL: sleep-onset latency
TST: total sleep time
WASO: wake after sleep onset
