# Supplementary material for: Effectiveness of Wearable Digital Therapeutics in Improving Sleep Outcomes Among Individuals With Insomnia: Systematic Review and Meta-Analysis of Randomized Controlled Trials
Source: J Med Internet Res. 2026 Sep 11;28:e93496. doi: 10.2196/93496 (PMC13568695; doi:10.2196/93496)
Supplement: Supplementary file 1 [file jmir-v28-e93496-s001.pdf]

**PubMed**	**548**
**#1**	"wearable electronic devices"[MeSH Terms] OR "wearable artificial intelligence"[Title/Abstract] OR "wearable computer"[Title/Abstract] OR "wearable device"[Title/Abstract] OR "wearable technolog*"[Title/Abstract] OR "wearable tracker"[Title/Abstract] OR "activity tracker"[Title/Abstract] OR "Fitbit"[Title/Abstract] OR "digital health"[Title/Abstract] OR "mobile health"[Title/Abstract] OR "telemonitoring"[Title/Abstract] OR "actigraphy"[Title/Abstract] OR "actiwatch"[Title/Abstract] OR "smartwatch"[Title/Abstract]
**#2**	"sleep initiation and maintenance disorders"[MeSH Terms] OR "sleep initiation"[Title/Abstract] OR "Sleeplessness"[Title/Abstract] OR "sleep disorders"[Title/Abstract] OR "Intrinsic"[Title/Abstract] OR "insomni*"[Title/Abstract] OR "difficulty falling asleep"[Title/Abstract]
**#3**	"randomized controlled trials as topic"[MeSH Terms] OR "RCT"[Title/Abstract] OR "controlled clinical trial"[Title/Abstract] OR "randomly assigned"[Title/Abstract] OR "Randomized"[Title/Abstract]
**#4**	#1 AND #2 AND #3

**Cochrane Library**	**2029**
**#1**	MeSH descriptor: [Wearable Electronic Devices] explode all trees
**#2**	(‘wearable artificial intelligence’ OR ‘wearable computer’ OR ‘wearable device’ OR ‘wearable technolog*’ OR ‘wearable tracker’ OR ‘activity tracker’ OR Fitbit OR ‘digital health’ OR ‘mobile health’ OR telemonitoring OR actigraphy OR actiwatch OR smartwatch):ti,ab,kw
**#3**	MeSH descriptor: [Sleep Initiation and Maintenance Disorders] explode all trees
**#4**	(‘Sleep Initiation’ OR Sleeplessness OR ‘Sleep Disorders’ OR Intrinsic OR insomni* OR ‘difficulty falling asleep’):ti,ab,kw
**#5**	MeSH descriptor: [Randomized Controlled Trial] explode all trees
**#6**	(RCT OR ‘Controlled Clinical Trial’ OR ‘randomly assigned’ OR Randomized):ti,ab,kw
**#7**	#1 OR #2
**#8**	#3 OR #4
**#9**	#5 OR #6
**#10**	#7 AND #8 AND #9

**Web of Science**	**788**
**#1**	TS=("Wearable Electronic Devices" OR "wearable artificial intelligence" OR "wearable computer" OR "wearable device" OR "wearable technolog*" OR "wearable tracker" OR "activity tracker" OR Fitbit OR "digital health" OR "mobile health" OR telemonitoring OR actigraphy OR Actiwatch OR smartwatch)
**#2**	TS=("Sleep Initiation and Maintenance Disorders" OR "Sleep Initiation" OR Sleeplessness OR "Sleep Disorders" OR Intrinsic OR insomni* OR "difficulty falling asleep")
**#3**	TS=("Randomized Controlled Trial" OR RCT OR "Controlled Clinical Trial" OR "randomly assigned" OR Randomized)
**#4**	#1 AND #2 AND #3

**Embase**	**1199**
**#1**	('wearable computer'/exp OR 'wearable electronic devices':ti,ab OR 'wearable artificial intelligence':ti,ab OR 'wearable device':ti,ab OR 'wearable technolog*':ti,ab OR 'wearable tracker':ti,ab OR 'activity tracker':ti,ab OR 'fitbit':ti,ab OR 'digital health':ti,ab OR 'mobile health':ti,ab OR 'telemonitoring':ti,ab OR 'actigraphy':ti,ab OR 'actiwatch':ti,ab OR 'smartwatch':ti,ab)
**#2**	('insomnia'/exp OR 'sleep initiation':ti,ab OR 'sleeplessness':ti,ab OR 'sleep disorders':ti,ab OR 'intrinsic':ti,ab OR 'insomni*':ti,ab OR 'difficulty falling asleep':ti,ab)
**#3**	('randomized controlled trial'/exp OR 'rct':ti,ab OR 'controlled clinical trial':ti,ab OR 'randomly assigned':ti,ab OR 'randomized':ti,ab)
**#4**	#1 AND #2 AND #3

**PsycInfo**	**271**
1	"Wearable Electronic Devices" OR "wearable artificial intelligence" OR "wearable computer" OR "wearable device" OR "wearable technolog*"OR "wearable tracker" OR "activity tracker" OR Fitbit OR "digital health" OR "mobile health" OR telemonitoring OR actigraphy OR Actiwatch OR smartwatch
2	"Sleep Initiation and Maintenance Disorders" OR "Sleep Initiation" OR Sleeplessness OR "Sleep Disorders" OR Intrinsic OR insomni* OR "difficulty falling asleep"
3	"Randomized Controlled Trial" OR RCT OR "Controlled Clinical Trial" OR "randomly assigned" OR Randomized
